# Genetic or pharmacological GHSR blockade has sexually dimorphic effects in rodents on a high-fat diet

**DOI:** 10.21203/rs.3.rs-3236045/v1

**Published:** 2023-10-18

**Authors:** Lorenzo Leggio, Andras Leko, Adriana Gregory-Flores, Renata Marchette, Juan Gomez, Janaina Vendruscolo, Vez Repunte-Canonigo, Vicky Chuong, Sara Deschaine, Kimberly Whiting, Shelley Jackson, Maria Cornejo, Mario Perello, Zhi-Bing You, Michael Eckhaus, Kim Janda, Barry Zorman, Pavel Sumazin, George Koob, Michael Michaelides, Pietro Paolo Sanna, Leandro Vendruscolo

**Affiliations:** National Institutes of Health; National Institutes of Health; National Institutes of Health; National Institutes on Alcohol Abuse and Alcoholism; NIH; National Institute on Drug Abuse; The Scripps Research Institute; National Institutes of Health; National Institutes of Health; National Institutes of Health; National Institute on Drug Abuse; Instituto Multidisciplinario de Biología Celular; Instituto Multidisciplinario de Biología Celular; National Institutes of Health; NIH; The Scripps Research Institute; Baylor College of Medicine; Baylor College of Medicine; National Institute on Drug Abuse; NIH; The Scripps Research Institute; National Institute on Drug Abuse

## Abstract

The stomach-derived hormone ghrelin regulates essential physiological functions. The ghrelin receptor (GHSR) has ligand-independent actions, therefore, *GHSR* gene deletion may be a reasonable approach to investigate the role of this system in feeding behaviors and diet-induced obesity (DIO). Here we investigated the effects of a long-term (12 month) high-fat (HFD) *versus* regular diet on obesity-related measures in global GHSR-KO and wild type (WT) Wistar male and female rats. Our main findings were that the *GHSR* gene deletion protects against DIO and decreases food intake during HFD in male but not in female rats. *GHSR* gene deletion increased thermogenesis and brain glucose uptake in male rats and modified the effects of HFD on brain glucose metabolism in a sex-specific manner, as assessed with small animal positron emission tomography. RNA-sequencing was also used to show that GHSR-KO rats had upregulated expression of genes responsible for fat oxidation in brown adipose tissue. Central administration of a novel GHSR inverse agonist, PF-5190457, attenuated ghrelin-induced food intake, but only in male, not in female mice. HFD-induced binge-like eating was reduced by inverse agonism in both sexes. Our results support GHSR as a promising target for new pharmacotherapies for obesity.

## Introduction

Obesity is a chronic disease that leads to serious health consequences, decreased life expectancy, and significant health care costs. In 2017–2018, obesity affected over 42% of adults in the United States^1^. There is a critical need to investigate novel pathways related to obesity and guide new treatment approaches^2^.

Ghrelin is an acylated 28 amino acid orexigenic hormone synthesized by endocrine cells, primarily located in the stomach, implicated in metabolism, reward, and food intake^3,4^. The *GHRL* gene encodes the post-translational formation of ghrelin by cleaving preproghrelin into proghrelin to be acylated by the ghrelin O-acyltransferase (GOAT)^5–7^. Ghrelin acts on the growth hormone secretagogue receptor (GHSR), which is widely expressed both in the central nervous system and periphery, including stomach, adrenal glands, and adipose tissue^8–10^. Recently, LEAP2 was identified as an endogenous GHSR antagonist that inhibits ghrelin’s effects and blocks constitutive GHSR activity^11^.

Circulating ghrelin levels fluctuate along with feeding states, increasing during fasting and decreasing during satiety. Both peripheral and central administration of ghrelin stimulate appetite, food seeking, and food consumption by linking peripheral signaling of the hormone to central modulation of feeding behaviors^12^. However, whereas ghrelin-related mechanisms in appetite, food intake, and reward have been established, this information has failed to translate into novel effective treatments for obesity, suggesting the need of further investigation on the role of ghrelin in obesity.

High-fat diets (HFDs), that range between 30 and 78% of total caloric content, can induce obesity in both rodents and humans^13–15^. Relevant to our study, food intake reduces postprandial plasma ghrelin levels, an effect that is blunted in obese people^16,17^. Not only is this dysregulation affecting feeding behaviors, but ghrelin also induces adiposity under HFDs^18^.

Although the orexigenic effects of ghrelin are regulated through the GHSR^19^, knockout rodents lacking the ghrelin peptide show little to no behavioral, physiological, or metabolic changes compared with wild-type (WT) controls^20,21^. These apparent discrepancies may be explained, at least in part, by some ligand independent actions of GHSR including its high intrinsic activity, even with the lack of ghrelin binding^22,23^. Therefore, deletion of *GHSR* gene provides a compelling proof-of-concept approach to investigate all the roles of this receptor in feeding behaviors and obesity. Toward this end, in the present work we investigated HFD *versus* regular diet in GHSR-KO male and female rats. These are CRISPR/Cas9-based global GHSR-KO Wistar rats, similar to which presented a phenotype of slightly reduced body weight and decreased food consumption under normal feeding (e.g., non-HFD) conditions, as *Zallar et al*. described earlier^24^. Those GHSR-KO male rats were also insensitive to ghrelin’s growth hormone (GH) secreting and orexigenic effects after systemic injection of ghrelin and had increased percentage of brown adipose tissue (BAT) in total body weight^24^.

Although interesting and promising, the experiments described above^24^ were limited to male rats. Expanding this work to both sexes is therefore important, even more so, given that ghrelin seems to play a role in sex-related differences in the physiology of eating^25^. Furthermore, in the previous work^24^, the rats were only fed a regular diet, and their body weight was followed and measured only throughout 13 weeks in the adulthood. To further study GHSR in a condition that better resembles what is observed in humans who have prolonged exposure to high-calorie food and develop obesity, we investigated the role of GHSR, in GHSR-KO and WT rats of both sexes, that were exposed to a HFD for 12 months. We used a comprehensive approach that involved metabolic, behavioral, endocrine, neuroimaging, and molecular measurements. We hypothesized that GHSR-KO male and female rats would be more resistant to HFD-induced behavioral and physiological changes compared with WT rats. Hence, they would also be more resistant to the HFD-led diet-induced obesity (DIO) phenotype.

HFD contributes to DIO also through inducing binge eating and hyperphagia^26^. GHSR constitutive activity in dopaminergic neurons is linked to hyperphagia induced by palatable foods, like HFD^27,28^. In the current study, in male and female mice, we administered the first GHSR inverse agonist, PF-5190457, which progressed to clinical development, and was found well-tolerable by oral administration in humans^29,30^. Furthermore, PF-5190457 reduced cue-induced food craving and food-seeking behavior in humans^30^. We hypothesized that, first, PF-5190457 will prove its pharmacodynamic efficacy by diminishing ghrelin-induced food intake, and second, it will reduce the HFD-induced hyperphagia. This pharmacological intervention may strengthen the translational potential of GHSR as a target against DIO and binge eating.

## Results

### Body Weight and Food Consumption

We measured body weight (BW, expressed in g) and food intake (expressed in g) twice weekly for 12 months, from the age of 2–4 months, to assess the effect of genotype on DIO. Rats were fed either a regular chow diet (Chow) with 24% protein, 58% carbohydrate, 18% fat (2.89 kcal/g) or a HFD with 20% protein, 20% carbohydrate, 60% fat (5.24 kcal/g).

**Figure 1:**
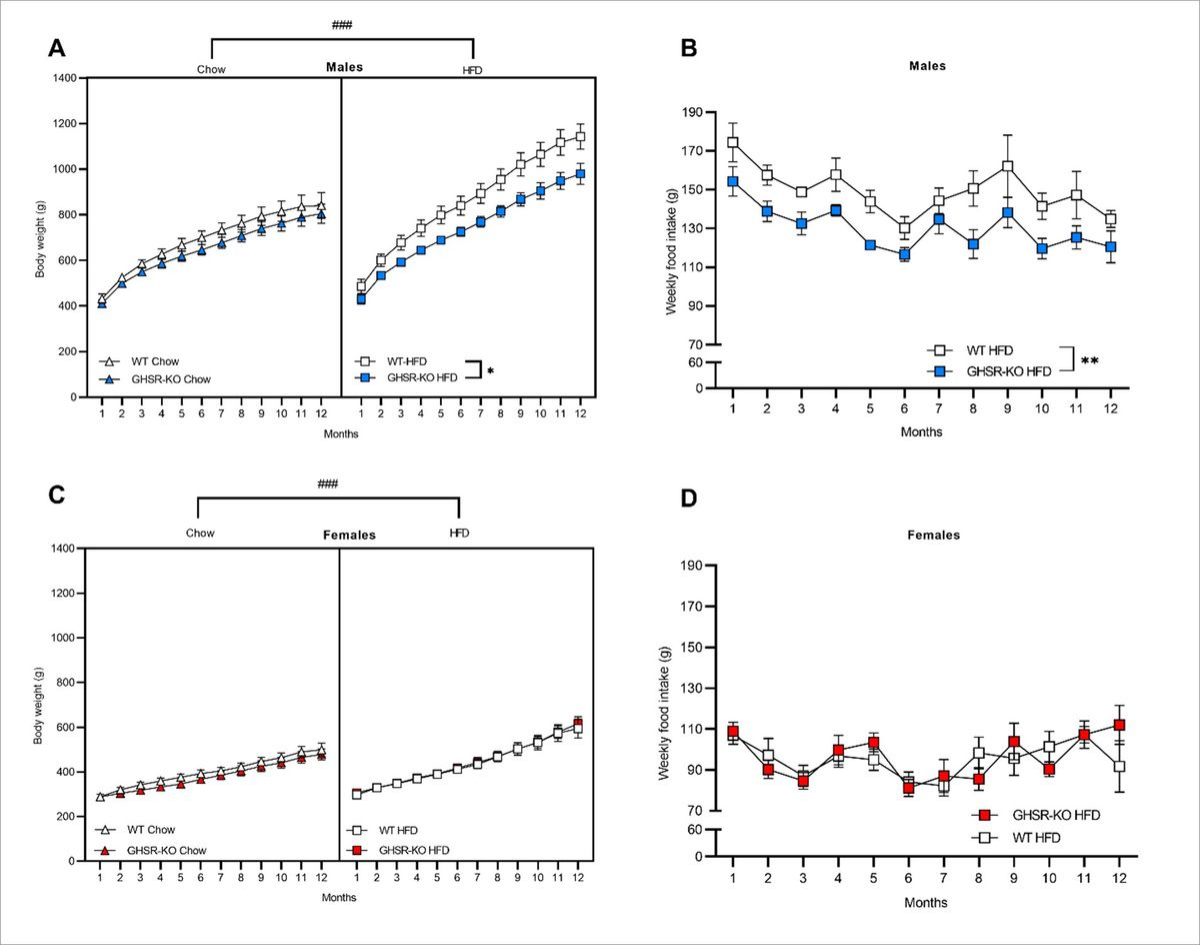
GHSR deletion has a protective effect against diet-induced obesity and decreases food intake in male, but not female rats exposed to a HFD. (**A**) GHSR-knockout (KO) male rats that had ad libitum access to a high-fat diet (HFD) weighed significantly less than wild-type (WT) male rats (*p < 0.05). Male rats that were maintained on a HFD weighed significantly more than male rats that were maintained on chow diet from months 2–12 (^###^p < 0.001). WT chow: *n* = 6. GHSR-KO chow: *n* = 7. WT HFD: *n* = 7. GHSR-KO HFD: *n* = 8. (**B**) GHSR-KO male rats on the HFD consumed significantly less food weekly than WT rats on the HFD (**p < 0.01, difference between WT and GHSR-KO rats regardless of time). (**C**) Regardless of genotype, female rats that were maintained on a HFD weighed significantly more than female rats that were maintained on chow diet from months 7–12 (^###^p < 0.001). WT chow: *n* = 10. GHSR-KO chow: *n* = 7. WT HFD: *n* = 6. GHSR-KO HFD: *n* = 10. (**D**) There was no difference between weekly food intake of WT and GHSR-KO female rats on HFD. Data are expressed as mean ± SEM.

For BW in males, with a 3-way repeated-measures (RM) ANOVA, we found significant effects of Time (F_1.32, 31.65_ = 377.72, *p* < 0.001), Genotype (F_1, 24_ = 7.66, *p* = 0.011; WT > GHSR-KO) and Diet (F_1, 24_ = 19.39, *p* < 0.001; HFD > chow), and a Time x Diet interaction (F_1.32, 31.65_ = 16.58, *p* < 0.001). *Post hoc* comparisons based on the Time x Diet interaction indicated that HFD-fed male rats weighted significantly more than chow-fed male rats from month 2 until the end of the study (*p* < 0.05; Fig. 1A). When analyzing HFD and chow-fed groups separately with 2-way RM ANOVA, the effect of Genotype was only seen in the HFD-fed animals, i.e., GHSR-KO rats’ weight was significantly lower compared to WT (*p* = 0.017; Fig. 1A).

For weekly food consumption in HFD-fed males, the 2-way RM ANOVA showed significant effects of Time (F_1.934, 25.14_ = 6.609, *p* < 0.005) and Genotype (F_1, 13_ = 10.81, *p* = 0.005), indicating that GHSR-KO rats on the HFD consumed less food compared to WT group (Fig. 1B).

For BW in female rats, 3-way RM ANOVA showed significant effects of Time (F_1.59, 46.04_ = 298.073, *p* < 0.001), Diet (F_1, 29_ = 6.734, *p* < 0.001), and a Time x Diet interaction (F_1.59, 46.04_ = 12.947, *p* < 0.001). *Post hoc* comparisons indicated that HFD-fed female rats weighted significantly more than chow-fed female rats in months 7–12 (*p* < 0.05; Fig. 1C), regardless of the genotype. Separate 2-way RM ANOVAs in different diet groups did not show any significant effect of Genotype in females (Fig. 1C).

For weekly food consumption in HFD-fed female rats, only a significant effect of Time (F_2.653, 37.14_ = 3.986, *p* < 0.001) was detected by the 2-way RM ANOVA (Fig. 1D).

The effects described above were unlikely due to differences in locomotion or anxiety-like behavior, as there were no genotype differences in an open field test or a novelty-suppressed feeding test (**Supplemental Figures S1 and S2**).

### Carcass Analysis

White adipose tissue (WAT), including visceral fat pads (gonadal, inguinal), intrascapular BAT, brain, liver, spleen, and adrenal glands, were collected and weighed after decapitation under deep isoflurane anesthesia. We analyzed the tissue weights normalized to the body weight and expressed them as % of total body weight.

In males, but not females we found a significant effect of Genotype (F_1, 24_ = 6.489, *p* < 0.05; GHSR-KO < WT) for the percent of gonadal WAT weight (**Supplemental Figure S3C**). We detected a significant effect of Diet on relative WAT weight, as HFD increased the percent of gonadal WAT weight in males and females (**Supplemental Figure S3C-D**), and the percent of inguinal WAT weight in males (**Supplemental Figure S3E**). A 2-way ANOVA showed an effect of Diet on the percentage of liver weight, indicating decreased liver weights in HFD-fed male and female rats compared with chow-fed rats, regardless of genotype (**Supplemental Table 1**). Given these results on liver weights, we further investigated hepatic lipid accumulation via a qualitative histological analysis of hematoxylin-eosin-stained liver slices. Livers of HFD-fed male and female rats showed mild-to-severe lipidosis, whereas livers of chow-fed rats in both sexes showed either no sign or only mild-to-moderate lipidosis (**Supplemental Figure S4**). For details see **Supplement.**

### Small Animal Positron Emission Tomography (PET) – Whole Brain

**Figure 2:**
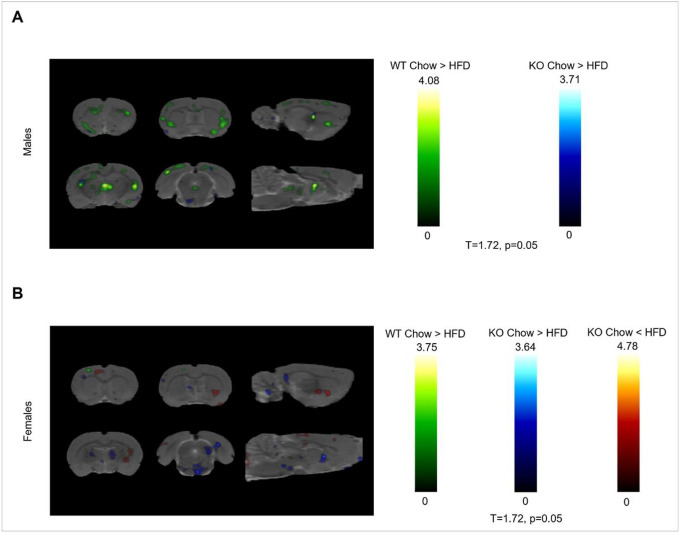
Small animal PET; effect of Diet. (**A-B**) Statistical parametric maps (T = 1.72; *p* < 0.05) of [^18^F] fluorodeoxyglucose (FDG) uptake as a function of Genotype and Diet (n = 6/treatment group) **(A)** Males: higher FDG uptake in chow vs. HFD in WT (green) and GHSR-KO (blue). **(B)** Females: higher FDG uptake in chow vs. HFD in WT (green) and GHSR-KO (blue); lower FDG uptake in chow vs. HFD in GHSR-KO (red).

A small animal PET scan with [^18^F] fluorodeoxyglucose (FDG) was used to measure glucose uptake in the whole brain, 50 weeks after initiation of Chow or HFD. First, the main effect of diet was analyzed separately in different sexes and genotypes. In male rats, HFD led to a decrease in FDG uptake, regardless of genotype. HFD-induced increase wasn’t detected. WT males were more affected by the diet, than GHSR-KO males (Fig. 2A). In females, HFD caused minor decreases in FDG uptake in WT rats, but marked decreases were detected in GHSR-KO rats. HFD led also to increases in multiple areas, distinct from decreases, in GHSR-KO females, meaning that GHSR-KO females were more affected by HFD (Fig. 2B).

**Figure 3:**
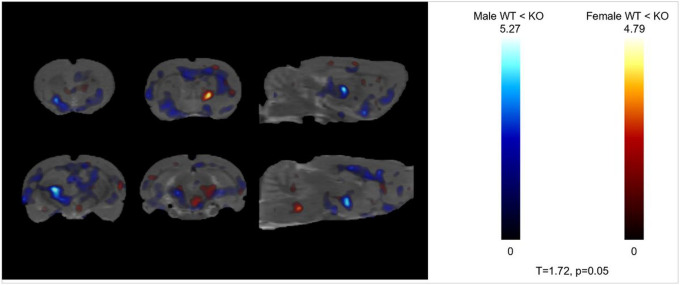
Small animal PET; effect of Genotype. Statistical parametric maps (T = 1.72; *p* < 0.05) of [^18^F] fluorodeoxyglucose (FDG) uptake as a function of Genotype and Sex, regardless of Diet. Males (blue): higher FDG uptake in KO vs. WT (n = 12 each). Females (red): higher FDG uptake in KO vs. WT (n = 12 each).

Second, the main effect of Genotype was analyzed separately in males and females. *GHSR* deletion causes increased FDG uptake throughout the brain, regardless of Diet. The effect was stronger in males and there were no decreases in uptake in either sex (Fig. 3).

### Brown Adipose Tissue (BAT) Thermogenesis

**Figure 4:**
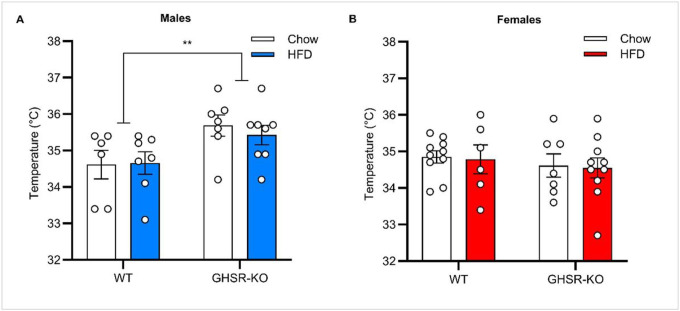
In the brown adipose tissue (BAT), GHSR KO leads to higher interscapular temperature in male, but not in female, rats. (**A**) GHSR-knockout (KO) male rats (*n* = 7 Chow, *n* = 8 HFD) had higher temperatures of the interscapular region compared with WT rats (*n* = 6 Chow, *n* =7 HFD), regardless of the Diet (**p < 0.01). (**B**) There were no significant differences between WT (*n* = 10 Chow, *n* = 6 HFD) and GHSR-KO (*n* = 7 Chow, *n* = 10 HFD) female rats fed a chow diet or HFD on temperature of the interscapular region. Data are expressed as mean ± SEM. Circles represent each individual rat.

To assess BAT thermogenesis, we determined surface body temperature of the interscapular region, where BAT is in rats. In male rats, a 2-way ANOVA indicated a significant main effect of Genotype (F_1, 24_ = 8.7, *p* < 0.01), with GHSR-KO male rats having higher temperatures than WT male rats, regardless of the Diet (Fig. 4A). In females, we did not detect significant effects for the temperature of the interscapular region (Fig. 4B).

### Endocrine Assays

We did not detect any effect of genotype in the glucose tolerance test (**Supplemental Figure S5**). In the insulin tolerance test, GHSR-KO males on HFD had slightly, but significantly, higher glucose levels during the test than WT males on the same diet (**Supplemental Figure S6**). In the terminal hormone analysis, GHSR-KO chow-fed male rats had higher progesterone levels compared to the WT group with the same diet, and HFD-fed group with the KO genotype (**Supplemental Figure S7H**). Aldosterone levels were increased in the chow-fed group (**Supplemental Figure S7I**). Leptin was higher with HFD, regardless of genotype (**Supplemental Figure S7J**). As an exploratory analysis, we investigated sex differences among the hormones directly related to the ghrelin system and detected significantly higher LEAP-2 levels in males, regardless of diet and genotype, compared with females (**Supplemental Figure S8**). For details see **Supplement.**

### RNA Sequencing (RNA-Seq) and Lipidomic Analyses of the Adipose Tissue in Males

**Figure 5:**
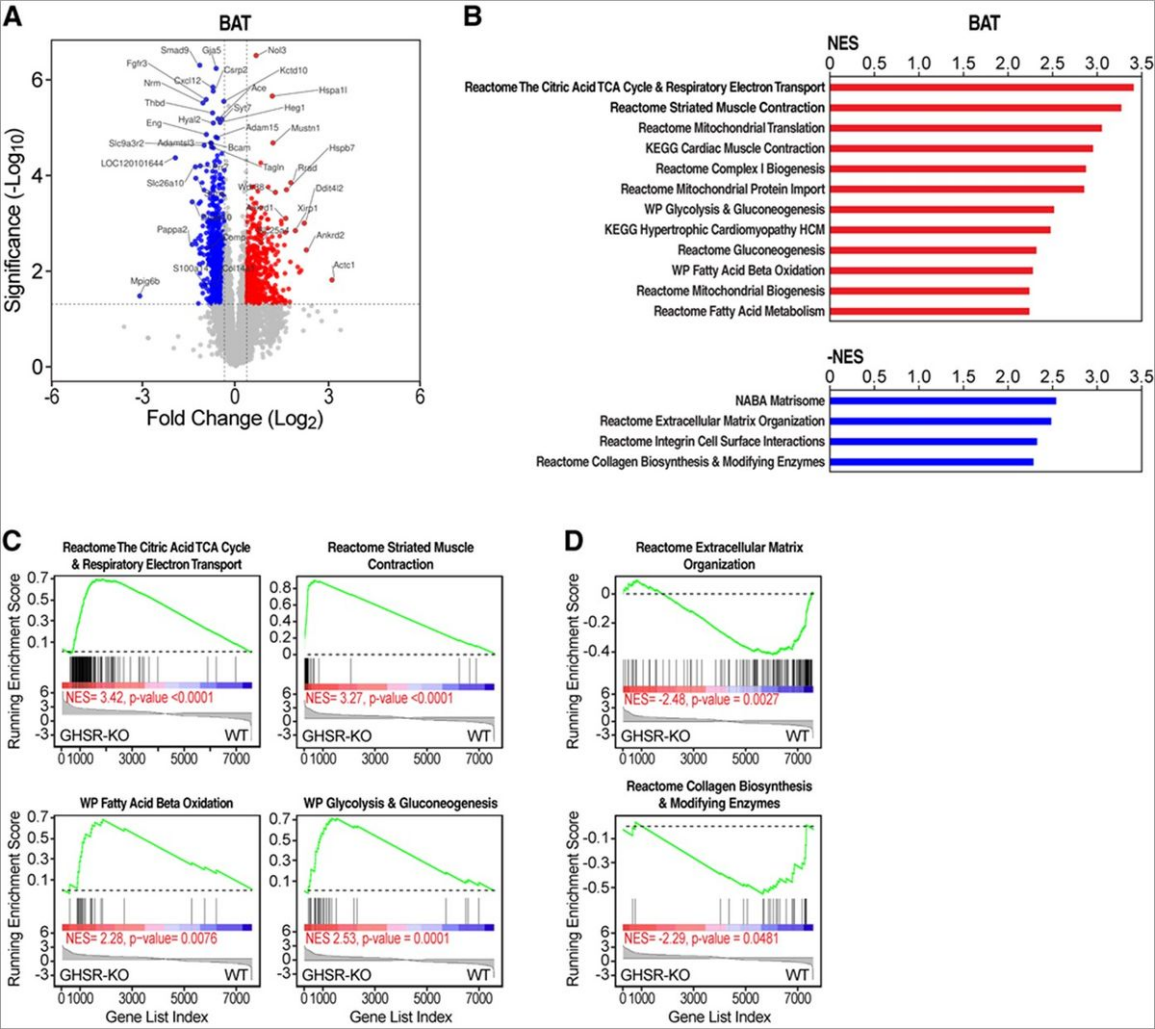
GHSR deletion induced a specific oxidative and myogenic gene signature in BAT of male rats. **(A)** Scatter (volcano) plot that depicts differentially expressed genes in the BAT of GHSR-KO rats on the HFD *vs*. WT rats on the HFD. X and Y axes, log2(Fold Change) and −log10(p-value). (**B**) Gene Set Enrichment Analysis (GSEA) pathway analysis for BAT of GSHR-KO on the HFD vs. WT rats on the same diet. GSHR-KO shows higher expression of genesets involved in mitochondrial function and energy metabolism, including TCA cycle, oxidative phosphorylation, electron transfer, fat metabolism, β-oxidation, and skeletal muscle-related genes (red bar graphs). Conversely, WT rats on the HFD showed higher expression of extracellular matrix-related genes (blue bar graphs) (genesets shown have *FWER p-values < 0.05*; overlapping genesets not shown; complete list is in **Supplemental Table 2**). NES; normalized enrichment score^31^. (**C**) GSEA plots of representative genesets upregulated in the BAT of GHSR-KO on the HFD vs. WT BAT on the same diet, which indicate upregulation of TCA cycle and respiratory electron transport; fat and glucose metabolism genes; and increased expression of BAT skeletal muscle-like gene program. (**D**) GSEA plots of representative genesets that indicate upregulation of extracellular matrix and collagen genes in WT BAT on the HFD vs. GHSR-KO BAT on the on the same diet. Changes in the expression of the pathway in the GSEA plots, such as the ones in **C,** are indicated by the asymmetric distribution of genes in the geneset (vertical bars) and of the line plot of the running NES toward the side of the condition indicated underneath: GHSR-KO or WT rats.

Gene expression was profiled by RNA-Seq in males because the effect of GHSR-KO was more prominent compared to females, in the experiments, described above. We used Gene Set Enrichment Analysis (GSEA) for pathway analysis^31^ in conjunction with genesets from the Molecular Signatures Database (MSigDB). Comparison of gene expression in the BAT of GHSR-KO *vs*. WT on the HFD by GSEA showed an upregulation of genesets in GHSR-KO that were representative of the tricarboxylic acid (TCA) cycle, oxidative phosphorylation, electron transfer, fat metabolism, β-oxidation, mitochondrial genes, glycolysis genes, and skeletal muscle-related genes (Fig. 5A–C and **Supplemental Table 2**); these findings were suggestive of increased fat oxidation. Conversely, the BAT of WT rats fed with the HFD showed greater expression of extracellular matrix, integrins and collagen biosynthesis, and modifying enzymes genes than the BAT of GHSR-KO rats on the same diet (Fig. 5A, B, D and **Supplemental Table 2**). The differential gene expression patterns of the BAT of GHSR-KO and WT were also evident in chow-fed rats (**Supplemental Figure S9A** and **Supplemental Table 3**); however, they were more pronounced in HFD-fed rats (**Supplemental Figure S9B** and **Supplemental Table 4**). Genesets related to TCA cycle, β-oxidation, mitochondrial genes, and skeletal muscle were also induced in WT rats by the HFD (**Supplemental Figure S9C** and **Supplemental Table 5**), but to a lower extent than in GHSR-KO rats (**Supplemental Figure S9D** and **Supplemental Table 6**).

**Figure 6:**
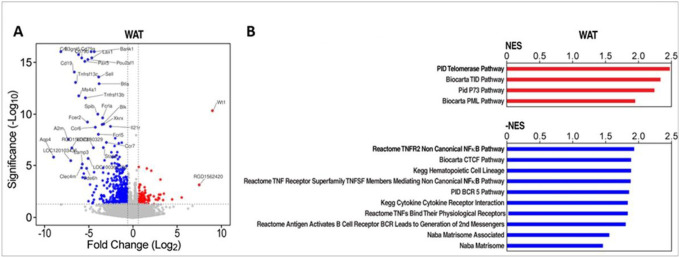
GHSR deletion induced telomerase activity and prevented against inflammatory gene expression in WAT of male rats. (**A**) Scatter (volcano) plot that depicts differentially expressed genes in the WAT of GHSR-KO rats on the HFD vs. WT rats on the same diet. X and Y axes, log2(Fold Change) and −log10(unadjusted p-value). X and Y values capped at 9 and 16, respectively. (**B**) Gene Set Enrichment Analysis (GSEA) pathway analysis for WAT of GHSR-KO vs. WT rats on the HFD suggest increased telomerase activity (red bar graphs) in GHSR-KO rats, while genesets with increased expression in WT indicate increased inflammation (blue bar graphs) (*NOM p-values < 0.05*). Complete lists of significantly differentially regulated genesets are in **Supplemental Table S6–9.** NES; normalized enrichment score^31^.

Gene expression analysis of the WAT of male WT rats on the HFD vs. GHSR-KO on the same diet showed increased expression of inflammation-related pathways, whereas pathways linked to telomerase activity were up-regulated in WAT of GHSR-KO rats on the HFD (Fig. 6 and **Supplemental Table 7**).

The results of the *lipidomic analysis* of WAT and BAT by mass spectrometry show that *GHSR* gene deletion significantly increased a subset of triglyceride (TG) species in the WAT in males on a chow diet. In BAT samples only two TG species were elevated in GHSR-KO males compared to WT (chow). Detailed description of the results is in **Supplement**.

### Central GHSR inverse agonism with PF-5190457 in WT and GHSR-KO mice

Notably, the studies reported here unmasked a dramatic sexually dimorphic phenotype as the deletion of GHSR protected against obesity in male, but not female, HFD-fed rats. Thus, we hypothesized that PF-5190457, the first GHSR antagonist/inverse agonist that has progressed to clinical development and is well-tolerable by oral administration in humans^29,30^, would also have sexually dimorphic effects in rodents.

**Figure 7:**
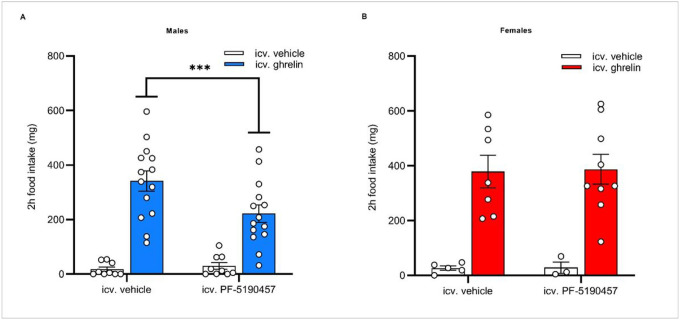
GHSR inverse agonism with PF-5190457 attenuated ghrelin-induced food intake in males, but not in females. (**A**) Ghrelin-induced food intake was reduced in male C57BL/6J mice, pretreated with icv. PF-5190457 (***p < 0.001; vehicle + vehicle, PF-5190457 + vehicle: n = 9, vehicle + ghrelin, PF-5190457 + ghrelin: n = 14). (B) In female C57BL/6J mice, icv. PF-5190457 pretreatment did not affect ghrelin-induced food intake (vehicle + vehicle, PF-5190457 + vehicle: n = 5, vehicle + ghrelin, PF-5190457 + ghrelin: n = 9)

First, we tested if intracerebroventricularly (icv.) administered PF-5190457 blocked icv. ghrelin-induced food intake in WT male and female C57BL/6J mice. We measured the amount of consumed chow pellets during 2 hours after icv. vehicle / PF-5190457 plus icv. vehicle / ghrelin administration. In males, 2-way RM ANOVA indicated a significant ghrelin x PF-5190457 interaction (F_1, 20_ = 11.67, *p* < 0.01), and post hoc comparisons indicated that PF-5190457 + ghrelin-treated mice consumed less chow than vehicle + ghrelin-treated mice (*p* < 0.001; Fig. 7A). In females, we did not see any ghrelin x PF-5190457 interaction (Fig. 7B). Ghrelin’s main effect on food intake was significant in both sexes, regardless of the pretreatment (males: F_1, 22_ = 41.72, p < 0.0001; females: F_1, 12_ = 28.27, p < 0.001; Fig. 7). In addition, we quantified the number of c-Fos immunoreactive (IR) cells in the hypothalamic arcuate nucleus (Arc) after the 2 hours food intake, in male and female mice treated with icv. ghrelin and PF-5190457. GHSR inverse agonist pretreatment results in lower number c-Fos-IR cells in ghrelin-treated male (*p* < 0.01) and female mice (*p* < 0.05) (**Supplemental Figure S11A-B**). Interestingly, PF-5190475 did not affect overnight food intake either in male or in female mice, compared to vehicle-treated animals (**Supplemental Figure S11C-D**).

**Figure 8:**
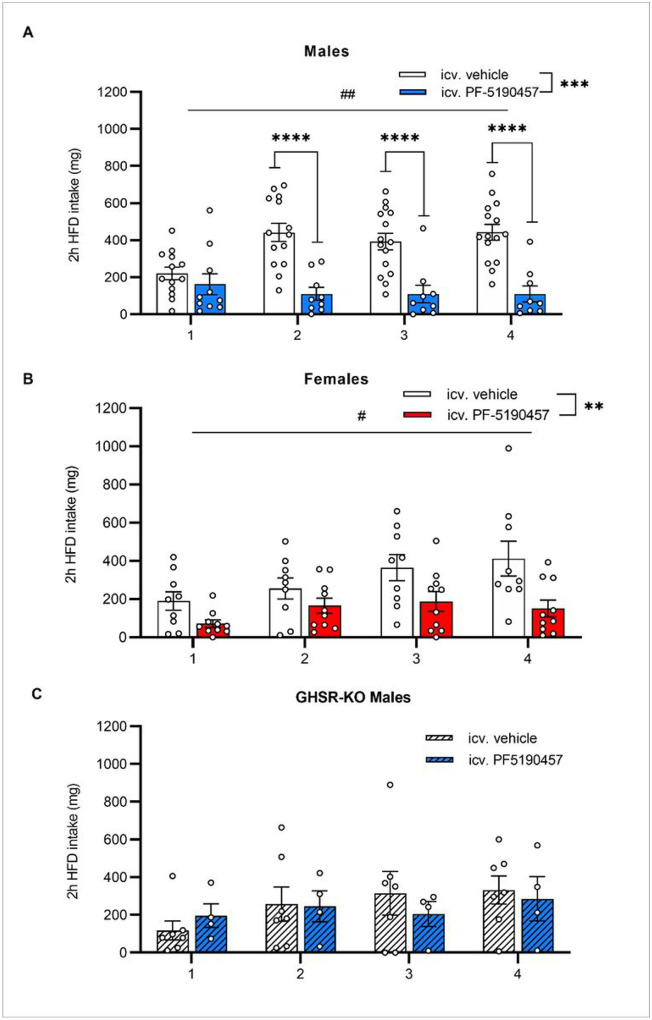
GHSR inverse agonism with PF-5190457 diminished HFD-induced binge-like eating in male and female WT mice, but not GHSR-KO male mice. (**A**) In male mice, repeated exposure to HFD led to an increase in 2-hour HFD-intake (^##^*p* < 0.01), but icv. PF-5190457 administration diminished this effect and reduced 2-hour HFD intake compared to icv. vehicle group on Days 2–4 (***p < 0.001; ****p < 0.0001; vehicle: *n* = 15, PF-5190457: *n* = 10). (**B**) In female mice, repeated exposure to HFD led to an increase in 2-hour HFD-intake (^#^*p* < 0.05), but icv. PF-5190457 administration diminished this effect and reduced 2-hour HFD intake compared to icv. vehicle group (**p < 0.01; vehicle: *n* = 9, PF-5190457: *n* = 10). (**C**) In GHSR-KO male mice, repeated exposure to HFD failed to increase 2-hour HFD-intake, and also icv. PF-5190457 did not have any effect.

Next, we tested the effect of icv. PF-5190457 on HFD-induced binge-like eating in satiated mice. We used a protocol with 2-hours HFD exposure, repeated throughout 4 consecutive days^27,28^. 2-way RM ANOVA showed a significant main effect of Time (males: F_3, 63_ = 4.391; *p* < 0.01; females: F_1.86, 31.55_ = 4.444; *p* < 0.05), meaning that repeated HFD-exposure induced binge-like eating. Main effect of Treatment (icv. PF-5190457 < vehicle) was also significant in both males (F_1, 23_ = 20.86; *p* < 0.001) and females (F_1, 17_ = 10.18; *p* < 0.01), while Treatment x Time interaction was only significant in males (F_3, 63_ = 12.97, *p* < 0.0001, Fig. 8A–B) meaning that PF-5190457-treated male mice consumed less HFD than vehicle-treated animals on days 2, 3 and 4 of the protocol (*p* < 0.0001). Finally, we exposed GHSR-KO male mice^32^ to the same HFD binge-like eating protocol and analyzed the effect of PF-5190457 administration. As expected, 2-way RM ANOVA detected no differences in HFD intake between PF-5190457- and vehicle-treated GHSR-KO male mice (Fig. 8C), and also HFD did not induce binge-like eating since the main effect of Time was not significant.

### Anti-Ghrelin Vaccine in Rats and Mice Exposed to HFD

Previous studies showed that peripheral sequestration of ghrelin by a ghrelin-specific vaccine blunted weight gain in non-obese male Swiss Webster mice^33^, non-obese male Wistar rats^34^, and pigs^35^. This vaccine is a putative target to manipulate GHSR signaling, but the potential sexual dimorphism of this effect was not investigated before. We examined the effects of a ghrelin-specific vaccine in the development of obesity using a HFD rat model (males) and subsequently we expanded this experiment to male and female mice as well. We detected only a modest IgG response after repeated vaccination in rats and mice, compared to similar studies^33,36–38^. Moreover, antibody plasma ghrelin binding affinity was muted in rats compared with an earlier study^34^. As such, full affinity analysis was not performed. Consistent with the limited immune response observed, our anti-ghrelin vaccine did not prevent weight gain, nor did it lower food consumption, in experiments with male HFD rats or with male and female Chow/HFD mice. See **Supplement.**

## Discussion

Our results present a multifaceted profile of how *GHSR* deletion modulates the effects of long-term (12-month) HFD in male and female rats. The main finding is that the deletion of *GHSR* protects against diet-induced weight gain and decreases food intake during HFD in male but not in female rats. The reduced food intake in our model was not related to changes in locomotor activity or anxiety-like behavior evaluated in the open field and novelty-suppressed feeding tests. All HFD groups, regardless of sex, accumulated more gonadal WAT, but in the males, WT rats had higher relative gonadal WAT weight (% of total body weight) than GHSR-KO. Using infrared thermography, we detected a higher body temperature of the interscapular region, where the BAT is mainly located, in GHSR-KO compared with WT rats in males but not females. Another novel finding of our study was that the HFD decreased brain FDG uptake in males of both genotypes, with a greater effect in WT than in GHSR-KO rats. In females, we observed the opposite, HFD attenuated FDG uptake more markedly, but also increased in certain brain areas in GHSR-KO rats. Furthermore, FDG uptake was increased in GHSR-KO male rats compared to WT, regardless of diet, an effect stronger in males than in females. Results of the RNA-sequencing in male rats showed upregulation of gene sets responsible for fat oxidation in the BAT of GHSR-KO and increased expression of connective tissue genes in WT, and these differences were more prominent in HFD. Furthermore, in the WAT during HFD, inflammation-related pathways were upregulated in WT compared to GHSR-KO. We also tested a therapeutically promising GHSR inverse agonist for the first time in both male and female mice. PF-5190457 is the only GHSR blocker which advanced to clinical trials and is well-tolerated in humans, and in our experiments icv. administration attenuated ghrelin-induced food intake interestingly only in male, but not female mice. The effect of GHSR inverse agonism on a more hedonic aspect of food intake, the HFD-induced binge-like eating, was not sex-dependent; icv. PF-5190457 reduced HFD intake in both sexes.

Our experiments demonstrate that diet-induced weight gain is attenuated in GHSR-KO male rats. The HFD in our model contained almost twice as many calories (5.24 *vs*. 2.89 kcal/g) as the regular diet, less carbohydrates (20% *vs*. 58%), and more fat (60% *vs*. 18%). The significantly reduced weight of GHSR-KO male rats in the HFD-fed group might result from decreased food intake compared to the WT male rats on the same diet. The initial characterization of our model^24^ used a regular diet for 34 weeks (*vs*. 48 weeks in the current study) in male rats only, and observed a small but significant reduction of body weight and food intake in the KO group. Of note, in the present study, we did not observe a decrease in body weight in rats on the regular diet, only on the HFD. In a previous 19-week study^32^ in male and female mice, the lack of GHSR reduced HFD intake and protected against HFD-induced weight gain compared to WT regardless of sex, whereas the lack of GHSR reduced weight in female but not in male mice fed a regular diet^32^. These findings indicate the complex interaction of sex and genetic modulation of the GHSR system in controlling food intake and body weight gain.

In the current study, GHSR deletion reduced normalized gonadal, but not inguinal, WAT weight (% of total body weight) regardless of diet. This finding may be explained by the lower expression of GHSR in the inguinal WAT than in gonadal WAT^39^. In rats, inguinal and gonadal fat pads are the main subcutaneous and visceral WAT depots, respectively^40^, suggesting that in the present study GHSR deletion reduced the ratio of visceral fat in the total body weight. The subcutaneous fat has a more beneficial metabolic profile compared to visceral fat in rodents and humans, by improving insulin action, longevity, protecting against ectopic fat deposition, lipotoxicity, and reducing tumorigenesis. The higher ratio of subcutaneous fat is also associated with lower triglyceride and higher high-density lipoprotein levels^40^. Deletion of GHSR may exert beneficial effects by reducing visceral (gonadal) WAT weight.

GHSR activity modulates the microstructure, gene-expression profile, and norepinephrine sensitivity of BAT, thereby affecting thermogenesis and energy expenditure. The number of mitochondria and the percentage of multilobular (brown) adipocytes is higher in GHSR-KO male mice^41^. GHSR expression in BAT increases with aging, leading to a thermogenesis reduction^39^. *GHSR* ablation attenuated the decline of thermogenesis, uncoupling protein-1 expression, and BAT’s norepinephrine content in male mice^39^. Consistent with this literature, we found that thermogenesis, measured by intrascapular temperature, was increased in GHSR-KO male rats, regardless of diet.

To gain a deeper understanding on the role of GHSR in BAT thermogenesis, we used RNA-seq to profile gene expression in the BAT in male rats. We found that *GHSR* gene deletion produced a BAT gene expression program characterized by the upregulation of skeletal muscle-related genes and other oxidative metabolism genes, such as tricarboxylic acid cycle and electron transfer genes, glycolytic genes, fat metabolism and β-oxidation genes, and mitochondrial genes. This effect of *GHSR* deletion became more pronounced during exposure to the HFD. Such a BAT gene expression profile has elements of the myogenic transcriptional signature that are dynamically regulated^42–44^. BAT and skeletal muscle, both of which contribute to the regulation of body temperature, have common cell lineages^42,43^. The skeletal muscle-like gene expression program in BAT and beige adipocytes is associated with heat generation^45^. Evidence shows that muscle-like actomyosin tensional response in BAT leads to calorie consumption that result in heat production and is induced by β-adrenergic activation^45^. Both BAT and skeletal muscle have high oxidative capacities and utilize glucose and fatty acids as substrates^46,47^. Glycolytic genes, fat metabolism, and β-oxidation genes were also increased in male rats by GHSR deletion. Increased glycolytic activity in beige fat in a myogenic state has also been associated with increased thermogenic capacity^48^.

WT male rats showed greater expression of extracellular matrix, integrins, and collagen genes. A late event in BAT gene expression response to HFD is the expression of connective tissue genes, including procollagen, procollagen cleavage, and remodeling genes, which takes place after the upregulation of the skeletal muscle-related gene expression program has subsided^44^. Thus, GHSR appears to direct the BAT gene expression program in male rats, both on the regular chow and on the HFD, possibly acting as a *switch* between a gene expression signature characterized by greater expression of the muscle-related gene expression program and one characterized by the expression of extracellular matrix, integrins, and collagen genes. The present results support GHSR as a therapeutic target that will implement the myogenic gene expression program in BAT, which has been proposed to be a therapeutic strategy for obesity and obesity-related metabolic disorders like type 2 diabetes^49^.

Gene expression analysis of the WAT of GHSR-KO male rats on the HFD vs. WT male rats on the same diet showed decreased expression of inflammation-related pathways in GHSR-KO male rats. As diet-induced obesity advances, angiogenesis can no longer keep up with the enlargement of adipose tissue. Pathological processes, such as hypoxia, may cause the dysregulation of adipokines, and adipocyte necrosis, leading to inflammation, fibrosis, and insulin resistance^50^. Confirming our findings, macrophage infiltration, as a marker of increased inflammation, was reduced in gonadal WAT of adipose tissue-specific GHSR-KO male mice^51^. Adipose tissue inflammation is reduced by interventions that improve fat oxidation and obesity^52–54^.

An additional finding of our study is the lipidomics analysis, based on an unbiased approach, to investigate the effect of HFD and genotype on the lipid profile of WAT and BAT. It is known that dietary fatty acid intake can cause changes in lipid composition. The HFD-fed rats in this study was high in saturated fatty acids, especially for triglyceride species^55^. We detected increased saturated and decreased unsaturated triglycerides in BAT and WAT due to HFD in both sexes. A recent study^56^ using an untargeted lipidomic approach for HFD-induced obesity in male rats showed a similar pattern in plasma, gonadal WAT, and liver. In our study, GHSR deletion affected the TG profile only in the WAT in males, leading to an increase in triglycerides with unsaturated fatty acids. This genotype-related difference was observed only with a regular diet, which can be explained by the robust decreasing effect of HFD on unsaturated triglycerides. We speculate that this effect of diet may have overshadowed the genotype effect.

The small animal PET highlighted differences in brain FDG uptake 50 weeks after initiation of a regular or high-fat diet in WT and GHSR-KO rats. HFD decreased brain FDG uptake, an observation consistent with early work in male Wistar rats^57^, male Sprague-Dawley rats^58^, and male C57BL/6J mice^59^. Yet, this work is novel not only because we investigated sex differences, but also for the length of HFD exposure, as previous studies measured FDG uptake only after 9^58,59^, or 16 weeks^57^. The brain glucose uptake by glucose transporters is also affected by HFD, as GLUT-4 protein levels were decreased after 9 weeks of HFD in male C57BL/6J mice^59^. GHSR gene deletion diminished the negative effect of HFD on brain FDG uptake in male rats, and increased FDG uptake regardless of diet. GHSR may affect the glucose transporters on neurons (GLUT-3) and on astrocytes (GLUT-2)^60^, as *Fuente-Martin et al*. showed that both acute and chronic central ghrelin administration decreased hypothalamic GLUT-2 and GLUT-3 protein levels in vivo in male Wistar rats, and GHSR gene deletion abolished this effect in *vitro*^60^. In female rats, we observed the opposite, namely HFD markedly decreased brain FDG uptake in GHSR-KO, but not in WT rats. This result is consistent with a recent study that showed no changes in brain FDG uptake after 16 weeks of HFD in female, but a decrease in male Wistar rats^57^. In addition, the main effect of Genotype (GHSR-KO > WT) on brain FDG uptake was less prominent in females.

This Wistar GHSR-KO model was previously characterized in males^24^. In mice, GHSR deletion was protective against HFD-induced weight gain in both sexes^32^. Sexual dimorphism of the ghrelin system has been described; for example, Sprague-Dawley female rats^61^ had higher plasma ghrelin levels, lower LEAP-2 expression in the liver, and higher GHSR expression in the hippocampus, amygdala, and LH. Gonadal hormones may be responsible for this sexual dimorphism given that ovariectomy diminished, and estrogen replacement restored the differences in ghrelin levels between males and females^61^. In our Wistar rats, we did not find any difference in ghrelin or desacyl-ghrelin levels between females and males, regardless of genotype, but LEAP-2 plasma concentration was higher in males. Furthermore, estrogen decreases the sensitivity to ghrelin’s orexigenic effects, as males and ovariectomized females were more responsive to peripheral and central ghrelin administration than intact or estrogen-treated ovariectomized Long-Evans rats^62^.

The terminal hormone analysis established that chow-fed GHSR-KO male rats had higher peripheral levels of progesterone compared to chow-fed WT males, and HFD-fed GHSR-KO males. To date, the effects of ghrelin and GHSR on progesterone levels was only investigated in female rats^63^. Ghrelin administration reduced progesterone levels throughout the estrous cycle in rats^64^. The effect of obesity was examined in human males, in that there was a negative correlation between plasma progesterone levels and body weight, body mass index, and waist circumference^65^.

We amended our comprehensive characterization of GHSR-KO Wistar rats with central pharmacological interventions targeting GHSR in male and female C57BL/6J mice. PF-5190457 is a promising compound, the first GHSR blocker ever tested in clinical trials. This inverse agonist was found being safe and tolerable, and reduced food-seeking in humans^30,36^. Our novel finding was that its effect on ghrelin-induced food intake is sex-dependent, can be observed only in males, similar to what we described here in GHSR-KO rats and DIO. Neuronal activation in the arcuate nucleus of the hypothalamus is the key target of ghrelin for the induction of food intake. PF-5190457 also reduced this activation as measured by the number of cFos-IR cells. We tested PF-5190457 in a HFD-induced binge-like eating paradigm, which models how palatable foods induce hedonic food intake and hyperphagia^26–28^. Icv. PF-5190457 was effective in both sexes, and we could confirm that this effect is GHSR-dependent. This founding highlights the role of GHSR constitutive activity in binge eating, which involve the GHSRs on dopaminergic neurons in the reward system^27,28^.

Study limitations include that our endocrine outcome was a terminal hormone analysis at a single timepoint, before euthanasia; and we could not measure the binding affinity of the antibody in mice treated with the anti-ghrelin vaccine. Of note, we employed, herein, a global KO model and this approach holds limitations related to long-term adaptations that may occur in constitutive KO rodents^66^. Nonetheless, this approach holds important value, especially as a proof-of-concept related to the role of GHSR in a translationally valid model of obesity and its relevance for genetic mutations in ghrelin systems in humans, such as single nucleotide polymorphism^67^. Of note, albeit in a different context and phenotype, i.e., people with alcohol use disorder (for a review, see *Farokhnia et. al*^68^), recent human work suggests that GHSR inverse agonism leads to a reduction in food-seeking behaviors^30^.

In conclusion, we demonstrated a protective effect of GHSR deletion in diet-induced obesity in male, but not female, rats, which shows a dramatic sexual dimorphism. Our pharmacological studies in mice also revealed that the GHSR blockade has stronger effects in males than in females indicating that the pharmacological intervention of the GHSR in humans requires a precise assessment of the sex effects. Our results indicate an important role for GHSRs in the regulation of body weight, food intake, energy homeostasis, adipose tissue, and brain activity, and constitute a promising target for medication development for the treatment of obesity.

## Materials and Methods

### Animals

WT and GHSR1-KO rats were obtained from the National Institute on Drug Abuse Intramural Research Program (NIDA IRP) breeding facility. Originating from a Wistar background, the initial characterization of these rats has been previously described^24^. A total of 61 rats were used. We used 28 male (13 WT; 15 GHSR-KO) and 33 female (16 WT; 17 GHSR-KO) rats that were 2–4 months old and weighed 150–350 g at the beginning of the study. Their body weight was recorded twice weekly for 12 months. All animals were single-housed and maintained under a 12 h/12 h light/dark cycle (lights off at 18:00) at 21°C ± 2°C. All procedures were conducted in accordance with the National Institutes of Health Guide for the Care and Use of Laboratory Animals and were approved by the NIDA IRP Animal Care and Use Committee, and IACUC of Multidisciplinary Institute of Cell Biology (IMBICE, National University of La Plata, Buenos Aires, Argentina).

### Diets

Rats were fed either a regular chow diet (Chow) with 24% protein, 58% carbohydrate, 18% fat (2.89 kcal/g; 2018 Teklad ENVIGO) or a HFD with 20% protein, 20% carbohydrate, 60% fat (5.24 kcal/g; Research Diets D12492, New Brunswick, NJ) for a total of 12 months (52 weeks). Animals were randomly assigned either Chow or HFD at the beginning of the study. For the duration of the study, rats had ad *libitum* access to food and water, except during periods of fasting required for the study procedures and behavioral testing described below.

### Body Weight and Food Consumption

Body weight (BW, expressed in g) and food intake (expressed in g per week) were recorded twice a week. Behavioral assessments (**Supplement**) and endocrine assays (**Supplement**) were also performed.

### Carcass Analysis

The rats had ad *libitum* access to food and water prior to euthanasia. Experimenters were blind to both the genotype of the rats and the diet that they received. The rats were weighed, and body measurements and temperature were taken. These measurements were followed by decapitation under deep isoflurane anesthesia. Brain, liver, spleen, and adrenal glands were collected and weighed. White adipose tissue (WAT), including visceral fat pads (gonadal, inguinal), and intrascapular BAT were dissected and weighed. The carcass analysis was adapted from a previously published protocol^69^ and was consistent with the carcass analysis described in the initial development and characterization of our GHSR-KO rat model^24^.

### Brown Adipose Tissue (BAT) Thermogenesis

At the end of the study (week 52), surface body temperature was determined using an infrared thermometer (FLIR E60). Rats were anesthetized, shaved at the rear base of the ears and center on the scapulae and positioned below the infrared thermometer. The thermometer was held at a focal length of 30 cm above the interscapular region where BAT is located underneath the skin of the rats.

### For the Small Animal PET, see Supplement Molecular RNA-Seq and Lipidomic Analyses of the Adipose Tissue

Given our previous work in non-obese GHSR-KO rats^24^ and the results of the BAT thermogenesis and body temperature (present study; see **Results**), adipose tissue from the carcass analysis was used for an unbiased hypothesis-generating analysis using gene expression and pathway analysis by RNA-seq (Supplement) and lipidomic analysis by mass spectrometry (**Supplement**).

### Ghrelin Vaccine in Rats and Mice Exposed to HFD

Methods of these experiments are in the **Supplement.**

For the Statistical Analysis, see Supplement.
